# Predictors of academic efficacy and dropout intention in university students: Can engagement suppress burnout?

**DOI:** 10.1371/journal.pone.0239816

**Published:** 2020-10-29

**Authors:** João Marôco, Hugo Assunção, Heidi Harju-Luukkainen, Su-Wei Lin, Pou-Seong Sit, Kwok-cheung Cheung, Benvindo Maloa, Ivana Stepanović Ilic, Thomas J. Smith, Juliana A. D. B. Campos

**Affiliations:** 1 William James Center for Research, ISPA–Instituto Universitário, Lisbon, Portugal; 2 Faculty of Education and Arts, Nord University, Bodø, Norway; 3 Department of Education, The National University of Tainan, Tainan, Taiwan; 4 Faculty of Education, University of Macau, Taipa, Macau, China; 5 Universidade Pedagógica, Maputo, Mozambique; 6 Department of Psychology, Faculty of Philosophy, University of Belgrade, Belgrade, Serbia; 7 Department of ETRA, Northern Illinois University, DeKalb, IL, United States of America; 8 Faculty of Pharmaceutical Sciences, Universidade Estadual Paulista, Araraquara, Brazil; IUMPA - Universitat Politecnica de Valencia, SPAIN

## Abstract

In this study we modelled possible causes and consequences of student burnout and engagement on academic efficacy and dropout intention in university students. Further we asked, can student engagement protect against the effects of burnout? In total 4,061 university students from Portugal, Brazil, Mozambique, the United Kingdom, the United States of America, Finland, Serbia, and Macao SAR, Taiwan participated in this study. With the data collected we analyzed the influence of Social Support, Coping Strategies, and school/course related variables on student engagement and burnout using structural equation modeling. We also analyzed the effect of student engagement, student burnout, and their interaction, on Academic Performance and Dropout Intention. We found that both student engagement and burnout are good predictors of subjective academic performance and dropout intention. However, student burnout suppresses the effect of student engagement on these variables. This result has strong implications for practitioners and administrators. To prevent student dropout, it is not enough to promote student engagement—additionally, and importantly, levels of student burnout must be kept low. Other variables such as social support and coping strategies are also relevant predictors of student engagement and burnout and should be considered when implementing preventive actions, self-help and guided intervention programs for college students.

## 1 Introduction

The term ‘‘Burnout” was first used to describe a Syndrome of exhaustion observed among mental health professionals [1]. Defined as a response to chronic interpersonal stressors in the workplace, the Burnout Syndrome comprises three dimensions: Exhaustion (EX), Cynicism (CY), and Inefficacy (INEF) [2]. Exhaustion is defined as the feeling of being overextended and depleted of cognitive, emotional, and physical resources. It is the central dimension of Burnout and represents individual stress. Cynicism is defined as a negative, callous, and detached attitude towards others. It is the interpersonal dimension of Burnout. Inefficacy is defined as feelings of incompetence, low productivity, and low achievement. It is the self-evaluative dimension of Burnout.

Several situational and individual factors have been associated with the prevalence of the Burnout Syndrome in the workplace [2, 3]. Situational factors include quantitative job demands (workload and time pressure), qualitative job demands (conflicting demands and lack of information), job resources (social support, information, control, feedback, participation, and autonomy), occupational characteristics (cognitive and emotional demands) and organizational characteristics (values implicit in organizational processes). The individual factors include demographic variables (age, gender, marital status, and level of education), personality characteristics (consciousness, hardiness, coping strategies, neuroticism, type-A behavior, and Jungian personality) and job attitudes (expectations).

With the publication of the MBI–General Survey (MBI-GS) it became possible to study the Burnout Syndrome outside of the human services [4–6]. Psychometric research with the MBI-GS demonstrated that the three-factor structure is invariant across various occupations [7, 8]. In recent years, the Burnout Syndrome has been measured in college students using the Maslach Burnout Inventory–Student Survey (MBI–SSi) [9–11]. Student Burnout (SB) can be defined as exhaustion due to study demands, a cynical and detached attitude towards the value of schooling, and feelings of academic inefficacy. Its three-factor conceptualization has been confirmed in multiple samples from different countries and study areas [9, 12–17] and its concurrent validity assessed against other measures of burnout [18].

In the workplace, burnout is a serious condition that has been linked with multiple physical and psychological conditions such as heart disease [19], depressive symptoms [20], and impaired cognitive performance [21]. Several parallels can be drawn from the work context to the academic context. The workload in the academic context corresponds to study demands (delivering assignments, preparing presentations, studying for tests, etc.). When high cognitive demands meet time pressure, a situation that is very likely to occur at the university level, students may experience severe chronic stress which, over time, can lead to Burnout [9, 22]. In student populations, the Burnout Syndrome has been linked with suicidal ideation [23], physical and psychological distress [14, 24], school dropout [17, 24], and poor academic performance [9]. Given the association of this Syndrome with these adverse conditions, the study of the possible causes and consequences of burnout in the academic context is of great importance to public health.

Burnout can lower academic engagement levels, such as class attendance, submission of schoolwork, and following teachers’ instructions [25, 26]. In this study, Student Engagement (SE) is conceptualized as a three-factor construct that includes behavioral, emotional, and cognitive dimensions [27–29]. Behavioral engagement is defined as students’ participation in classroom tasks, student conduct, and participation in school-related extracurricular activities. Cognitive engagement is defined as the students’ investment and willingness to exert the necessary efforts for the comprehension and mastering of complex ideas and difficult skills. Emotional engagement is defined as attention to teachers’ instructions, perception of school belonging, and beliefs about the value of schooling. Engagement and burnout can be seen as two poles of an engagement-burnout spectrum, where burnout is seen as the erosion of engagement [3]. However, this conceptualization ignores the fact that people with low levels of burnout may not be engaged in their work [9]. If we consider that SE and SB, although negatively correlated, are not conceptual opposites, the interaction between the two variables becomes possible. In this view, SE and SB can be both a cause and a consequence of each other: high levels of burnout can lead to a decrease in engagement; high levels of engagement can be a protective factor against burnout. Although these two dimensions interact, we expect student burnout to be negatively correlated with student engagement, academic performance, and positively correlated with dropout intention. In this research, we also anticipate the potential effects of coping strategies, social support, course expectations, teacher competence, and the need for medication as explanatory variables for student engagement and burnout.

Coping strategies can be defined as efforts to avoid or decrease threats and reduce associated stress [30, 31]. In general, coping strategies can be divided into two categories: active or positive coping (seeking information, seeking help, seeking social support, planning, and accepting or reframing problems with humor or faith) and passive or negative coping (disengagement, self-distraction, denial, self-blame, substance abuse, venting, etc.), although other divisions are possible, such as emotion-focused coping strategies and task-focused coping strategies [32, 33]. Passive coping is often maladaptive because it employs emotion-oriented strategies such as rumination or excessive emotional responses [34]. Maladaptive coping has been previously linked to student burnout [35]. Active and passive coping strategies are associated with a tendency to have an external or internal locus of control. People who have a tendency for external attributions believe that outcomes are not dependent upon their actions but rather the result of powerful others or due to chance [36]. People with an internal attribution style believe that outcomes are the result of their own ability and effort. A passive and avoidant coping style is often associated with external attributions, while an active confrontative coping style is often associated with internal attributions. A passive and avoidant coping style is associated with low levels of hardiness (involvement in daily activities, sense of control over events and openness to change), poor self-esteem and external locus of control, which typically constitute the profile of a stress-prone individual [2]. A passive coping style may be detrimental to students’ attitudes towards academic challenges as it incentivizes students to avoid their problems. For instance, students may believe that an existing problem is external and that there is nothing that they can do to improve their situation. This attitude can lead students to fall into a downward spiral of academic disengagement that leads them further and further away from meeting their academic demands. Our expectation, to be tested in this study, is that a negative coping style is positively associated with burnout and negatively associated with student engagement.

It is important to note that academic demands are not the only stressor that students must deal with at the university level. Other challenges include peer pressure and competition, limited socioeconomic power, and distance from home and family, among others [37–39]. One important predictor of perceived stress among college students is social support [40–43]. Student social support can be defined as having good relationships with family members, friends, colleagues and professors. Students with good social support feel loved, esteemed, and valued by people around them. Social support is important because, in addition to the emotional support and instrumental assistance, it reaffirms the validity of the students’ membership in the academic environment, building on core motivational values. This network of personal relationships provides students with the emotional, financial, and informational support needed to prevent and endure the high levels of stress experienced in the academic context [44]. We expect social support to be negatively associated with student burnout and positively associated with student engagement.

Finally, we focus on course expectations, students’ evaluation of teacher competence, and the need for medication as predictors of student engagement and burnout. Course expectations are a key motivational factor that promotes engagement [45] and their absence is a risk factor for student burnout [46]. Teacher competence is another central motivational factor that promotes student engagement [47]. Lastly, the need for medication for medical reasons has substantial prevalence among university students which may have serious effects on student engagement [48].

**Fig 1:**
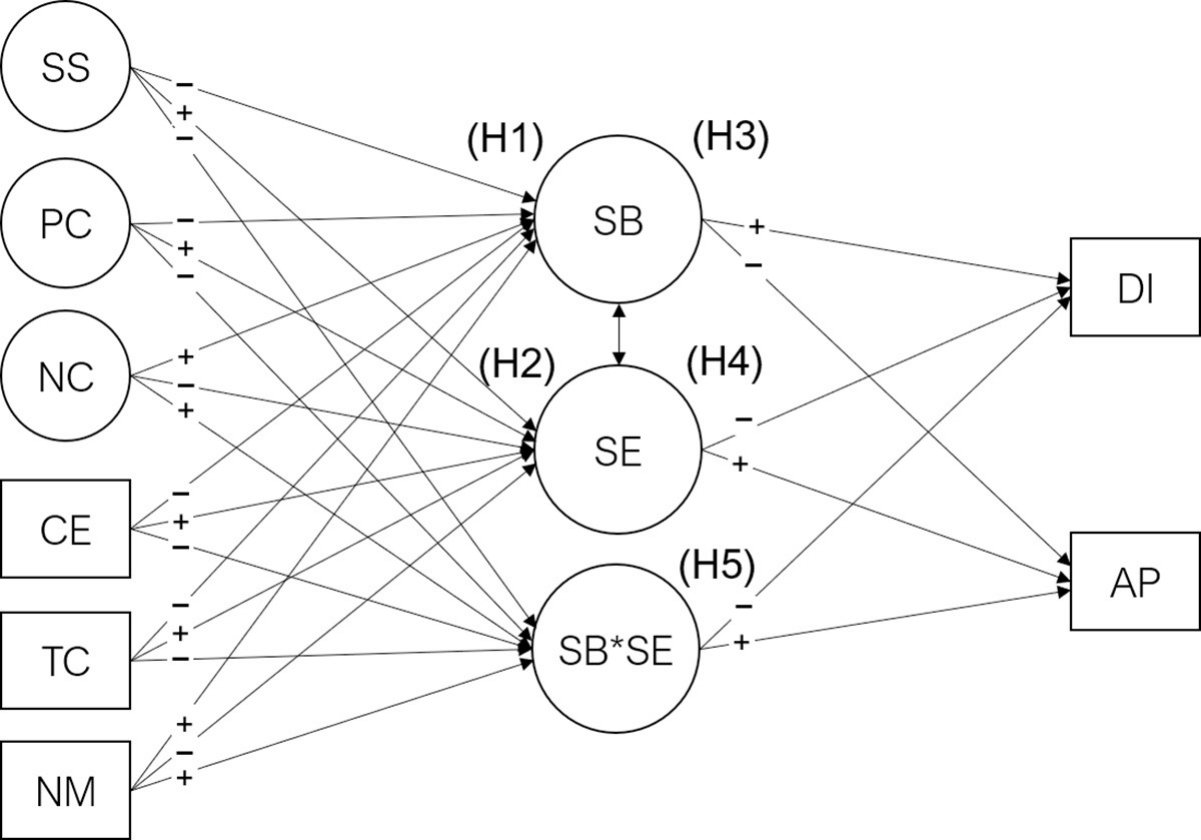
Student burnout and engagement mediation and moderation model. CE = Course Expectations, TC = Teacher Competence, NM = Need for Medication, PC = Positive Coping, NC = Negative Coping, SS = Social Support, SB = Student Burnout, SE = Student Engagement, SB*SE = Interaction between SE and SB, DI = Dropout Intention, AP = Academic Performance. For clarity, direct effects of the exogenous variables (CE, TC, NM, PC, NC, SS) on the endogenous variables (DI, AP) are omitted. Plus signs indicate positive expected effect and minus signs indicate negative expected effect. See further text for hypothesis (H1 to H5).

In this research, our first aim is to assess the possible causes and consequences of student engagement and burnout. We expect that social support, coping strategies, course expectations, teacher competence, and the need for medication will significantly predict student burnout (H1) and engagement (H2). We also expect student burnout (H3) and student engagement (H4) to be significant predictors of subjective academic performance, and dropout intention. Our second aim is to assess the moderation effect that student engagement has on the relationships between student burnout and each of the criterion variables. We expect that student engagement will act as a protective factor against the effects of student burnout (H5). The expected relationships between constructs can be found in the model path diagram in Fig 1. Our final aim is to test the structural invariance of this model across different genders, areas of study, and countries/regions (H6).

## 2 Materials and methods

### 2.1. Procedures

An online questionnaire was created using the Qualtrics platform. The order of appearance of the scales were randomized between participants and only fully completed questionnaires were considered for further analysis. At the end of the questionnaire, participants answered a series of sociodemographic and academic-related questions. The survey was designed to take 15 minutes to complete. The content, objectives, duration, risks, data policy, ethics approval, and contacts were provided at the start of the questionnaire. The study was properly validated by ISPA-Instituto Universitário Ethics Commission (Process: I/017/02/2019) and by the Northern Illinois University International Review Board (decision 1504/2014; FWAA00004025). Informed consent was obtained from all individual participants in the study. All procedures performed in studies involving human participants were in accordance with the ethical standards of the Institutional Ethics Research Committee and with the 1964 Helsinki declaration and its later amendments or comparable ethical standards.

### 2.2. Participants

The minimum sample size required for confirmatory factor analysis for the USEI measurement model (the model with the larger number of parameters to estimate) was determined by Monte‐Carlo simulation as suggested by Brown [49] with criteria defined by Muthén and Muthén [50]: (a) Bias of parameters estimates smaller than 10%; (b) 95% confidence intervals coverage larger than 91% and (c) percentage of significant coefficients (power) larger or equal to 80%. Mplus software (version 8, Muthén & Muthén, Los Angeles: CA) was used for simulations with the second-order confirmatory factor analysis model using factor loadings from the original USEI study [25]. One thousand samples of size 100, 200 and 300 were simulated. A minimum sample size of 200 was shown to be enough to attain bias below 1% for both point estimates and standard errors of the parameters; 99% confidence interval coverage greater than 95%, and minimum power of 90%. However, to ensure that the study sample, which was non–probabilistic, would capture a large amount of the normative population variance we set the sample size at a minimum of 300 students per country/region corresponding to 20 participants per item of the full SEM model as suggested by Marôco [51]. A non-probabilistic convenience sampling method was chosen to achieve this sample size. Co-authors in each country distributed the online survey to students and student associations in each country/region, directly in class, via e-mail and online social media platforms. We estimate that between 16 thousand and 30 thousand university students were contacted to participate in this study across all countries surveyed. The exact number is difficult to estimate because online link to the survey were publicly available on different online platforms. However, given that we registered a total of 15,172 survey visits, which lead to‬ 4,061 completed surveys, we estimate a minimum reach of 50.0% and a completion rate of 26.8%.

The study sample was composed of 4,061 university students (ages ranging from 16 to 70 years; M = 23.2; SD = 5.6; Mdn = 21) from Portugal (1,067), Brazil (424), Mozambique (413), United Kingdom (314), United States of America (316), Finland (356), Serbia (409), Macao SAR and Taiwan (762). The typical participant was female (60%), pursuing a bachelor’s degree (74%) in human and social sciences (51%) in a public (88%) university (80%), living with their family (54%), which financed their studies (56%). See Table 1 for further characterization of the study sample.

**Table 1:** Demographic and academic variables by country.

Country	PT	BR	MZ	UK	USA	FIN	SER	TW&MO
Sample Size	1,067	424	413	314	316	356	409	762
Age (Mean)	22.9	23.3	26.3	22.6	21.9	26.2	22.0	22.3
Age (Median)	21.0	22.0	25.0	21.0	20.5	24.0	22.0	21.0
Age (S.D.)	6.7	5.3	6.8	5.3	4.3	7.8	2.2	5.4
Women (%)	65.3	43.4	62.2	47.1	34.8	62.1	83.9	66.1
Public School (%)	90.3	85.1	97.3	86.0	81.0	94.4	96.3	79.8
Human and Social Sciences (%)	33.4	39.2	71.9	49.0	37.0	56.5	53.3	66.5
Exact Sciences (%)	29.6	38.7	11.4	36.0	50.0	32.3	20.8	22.0
Biological Sciences (%)	9.7	9.2	12.1	8.9	8.2	6.7	4.6	6.3
Health Sciences (%)	27.4	13.0	4.6	6.1	4.7	4.5	15.9	5.1
Dropout Intention (Mean)	1.2	1.6	0.6	1.1	1.0	0.8	0.6	0.5
Academic Performance (Mean)	2.5	2.5	2.7	2.7	2.8	2.6	2.9	2.4
1st Year (%)	17.7	13.6	39.5	26.3	20.1	15.6	21.3	21.9
2nd Year (%)	18.6	18.1	20.1	20.5	29.4	15.6	21.8	18.4
3rd Year (%)	21.9	20.3	8.7	14.1	36.3	20.8	23.3	28.4
4th Year (%)	15.6	24.1	26.6	22.9	4.3	17.3	28.7	19.1
5th Year (%)	7.0	19.8	3.9	4.7	3.0	28.3	1.3	5.2
6th Year (%)	4.1	1.4	0.0	4.0	1.3	0.9	1.8	3.7
7th Year (%)	7.3	1.7	0.7	2.0	1.7	0.9	0.8	0.9
8th Year (%)	6.8	0.7	0.5	2.4	2.6	0.3	0.0	1.6
9th Year (%)	0.6	0.2	0.0	3.0	1.3	0.3	1.0	0.5
10th Year (%)	0.4	0.0	0.0	0.0	0.0	0.0	0.0	0.3

Note: The number of years in this table reports the number of successful transitions from the first year of bachelors up to the doctorate. PT–Portugal; BR–Brazil; MZ–Mozambique; UK–The United Kingdom; USA–United States of America; FIN–Finland; SER–Serbia; TW&MO–Taiwan and Macao.

### 2.3. Measurement instruments

An online questionnaire was constructed containing the following psychological measures: the Maslach Burnout Inventory adapted to students, version MBI-Student Survey (MBI-SSi) [51], the Social Support Satisfaction Scale (ESSS) [41, 44], the University Student Engagement Inventory (USEI) [25], and the BriefCOPE Inventory adapted to university students [52]. Additionally, participants responded to a set of demographics, personal, and academic queries. Five versions of the questionnaire were used in this study: Portuguese (Portugal, Brazil, and Mozambique), English (the United Kingdom, the United States of America, and Finland), Serbian (Serbia), and traditional Chinese (Macao and Taiwan). Measuring instruments were translated by coauthors via group consensus and translated back into the original language for double-checking.

#### 2.3.1. Maslach Burnout Inventory—Student Survey (MBI-SSi)

The Maslach Burnout Inventory—Student Survey with the efficacy dimension reversed (MBI-SSi) [51] was used to measure student burnout levels. Student burnout is conceptualized as a second-order construct reflected in the first order exhaustion, cynicism, and inefficacy dimensions. The MBI-SSi consists of 15 self-report items rated along 7 ordered response categories from ‘0—never’ to ‘6- every day’. In its original formulation [9] the Efficacy dimension of the MBI-SS has positively worded items while Exhaustion and Cynicism consist of negatively worded items. Here we use a version of the MBI (MBI-SSi) [51] where the items in the Efficacy dimension are negatively worded to give rise to the Inefficacy (INEF) dimension. Psychometric analysis of data collected with both the MBI-SS and MBI-SSi revealed that reliability and construct validity of the MBI factors were improved in the MBI-SSi [51].

#### 2.3.2. University Student Engagement Inventory (USEI)

The University Student Engagement Inventory (USEI) [25] was used as a measure of student engagement. In the USEI, student engagement is conceptualized as a second-order factor construct that is reflected by behavioral, emotional and cognitive dimensions. The USEI consists of 15 self-report items rated using ordered response categories from ‘1-never’ to ‘5-always’. Each of the three first-order factors consists of five items. The USEI has been assessed previously for factorial validity and reliability [25] and measurement invariance across genders and areas of study [53] but only for Portuguese speaking students.

#### 2.3.3. Social support (ESSS)

The Satisfaction with Social Support (ESSS) [41, 44] was used to measure student social support. Social support is conceptualized as a second-order construct reflected by four first-order dimensions (social activities, satisfaction with family, intimacy, and satisfaction with friendships). The ESSS consists of 15 self-report Likert items with response options ranging from ‘1 –Totally disagree’ to ‘5- Totally agree’.

#### 2.3.4. Coping strategies (BriefCOPE)

The BriefCOPE version of the COPE Inventory [54] was used as a measure of coping strategies. BriefCOPE is a self-completion tool consisting of 28 self-report items grouped in 14 scales, where each item is rated using ordered response categories ranging from ‘0—I never did this’ to ‘4 –I always do this’. Based on conceptual similarity and correlation patterns, positive and negative coping second order factors were created. The positive coping factor consisted of following factors: active coping, positive reframing, planning, and instrumental support. The negative coping factor consisted of following factors: self-blame, denial, behavioral disengagement, and substance use.

#### 2.3.5. Socio-demographic and academic-related questions

The demographic variables assessed were gender, age, region, household, and financial support. The self-reported academic variables were the area of studies [examples of the different areas of study were provided for respondents guidance as follows: Human and Social Sciences (e.g., Psychology, Law, Sociology, Economics, Education…); Exact Sciences (e.g., Math, Statistics, Physics, Chemistry,…); Biological Sciences (e.g., Biology, Agronomy, Environment,…) and Health Sciences (e.g. Medicine, Nursing,…)], type of degree (bachelor, master, doctorate), type of school (public/private university), course expectations, teacher competence, academic performance, need for medication, dropout intention, the total number of classes and number of failed classes.

From the socio-demographic and academic-related variables, we selected course expectations, teacher competence and need for medication as predictor variables, academic performance, and dropout intention as criterion variables. Variables were measured using a 4 or 5-point Likert/rating scale. Course expectations were measured with the item "Concerning your initial expectations this degree is:" with answers ranging from "Much worse" to "Much better". Teacher competence was measured with the item "In general terms how do you classify your professors?" with answers ranging from "Very incompetent" to "Very competent". Subjective academic performance was assessed with the item "How do you classify your performance in this degree?" with answers ranging from "Bad" to "Excellent". Dropout intention was measured with the item "Have you thought of dropping out of college?" with answers ranging from "Never" to "Very frequently". Need for medication was measured with the item "Do you need, or have you needed any sort of medication because of your college studies?" with answers ranging from "Never" to "Always".

### 2.4. Data analysis

#### 2.4.1. Measurement models

A series of Confirmatory factor analysis (CFA) were conducted to evaluate the goodness-of-fit of the measuring models of Burnout, Engagement, Social Support, Positive Coping, and Negative Coping factors with the lavaan package [55] of the open-source R statistical system [56]. Factors’ reliabilities were estimated with McDonald’s Omega (for first order factors) and Omega L2 (for second order factors) using the reliability functions provided by the SemTools package [57] for R. We opted to not impute missing values because most incomplete questionnaires did not complete 10% of the questions.

#### 2.4.2. Structural equation modeling

After assuring good psychometric properties of the measurement model, four Structural Equation Models (SEM) were created with the lavaan package [55] for R. All models featured three exogenous observed predictors (Course Expectations, Teacher Competence and Need for Medication), three exogenous latent predictors (Social Support, Positive Coping, and Negative Coping), and two endogenous criterion variables (Academic Performance and Dropout Intention). The first model featured student burnout as a mediating factor between predictor and criterion variables. The second model featured student engagement as a mediating factor between predictor and criterion variables. The third model featured both burnout and engagement as mediating factors. Finally, the fourth model was built upon the third model adding a moderation effect of burnout and engagement on the criterion variables. The moderation effect of student burnout and engagement was a latent variable indicated by the paired centered products of the items from the original factors to reduce multicollinearity effects [58–60].

Because some items had unanswered categories, it was not possible to use WLSMV for categorical items to test the thresholds’ invariance of the models. However, when items have five or more points and a distribution that is not severely non-normal (absolute skewness and kurtosis values below 7 and 3, respectively), Pearson correlations estimate the associations between variables reasonably well [58, 61]. Thus, SEM and invariance analyses were carried out using robust maximum likelihood estimation (MLR) implemented in lavaan [55]. This method accounts for deviations from the normal distribution of the items on the estimation of the parameters’ standard-errors and model fit indices. Measurement errors of items belonging to the same factor were correlated when their modification indices were greater than 11 (p < .001) [58] and the suggested theoretical association could be justified.

The following goodness-of-fit indices for both confirmatory factor analysis and structural equation modeling were used: χ^2^(df) (Chi-Square Statistic and degrees of freedom), CFI (Confirmatory Fit Index), TLI (Tucker-Lewis Index), RMSEA (Root Mean Square Error of Approximation) and SRMR (Standardized Root Mean Square Residual). Because robust maximum likelihood estimation was used, the robust scaled versions of the indices were used. Model fit was considered adequate if CFI and TLI values were above .90 and RMSEA and SRMR values were below .06 and .08 respectively Acceptable reliability was assumed for Omega (ω) and Omega L2 larger or equal than .7 [58, 62].

#### 2.4.3. Model invariance by gender, area of studies and country/region

Model invariance was tested for gender, area of study, and country/region, following the recommendations of Millsap and Yun-Tein (2004), and Wu and Estabrook (2016) [63, 64]. A configural model was created where parameters were freely estimated between groups. This model served as a baseline for further invariance testing. Six nested models were created where factor loadings (loadings), items’ intercepts (intercepts), factor intercepts (means), second-order factor loadings (regressions), structural coefficients (structural) and residual variances (residuals) were sequentially fixed between groups and tested. Fit indices of the nested models were assessed to probe for invariance. Model invariance is generally assessed using the Cheung and Rensvold ΔCFI criterion (|ΔCFI| < .01) [65]. For large sample sizes (thousands of participants) and large number of groups, Rutkowski and Svetina [66] suggest that slightly more liberal criteria around .020 and .030 for |ΔCFI| and |ΔRMSEA| respectively, should be adopted for metric invariance. If less restrictive models of invariance fail, further sequential testing of more restrictive models is not recommended [58, 63, 64]. When factor loadings and regression coefficients were invariant between groups, but not intercepts, weak factorial or metric invariance was assumed. Metric invariance means that the contribution of each item to the factor remains constant across different groups. When factor loadings and intercepts were invariant across groups, strong or scalar invariance was assumed. Scalar invariance enables comparisons between group means [63]. When factor loadings, intercepts, second-order factor loadings (regressions), and structural regression coefficients were invariant across groups, full invariance was assumed [63, 64].

## 3 Results

### 3.1. Measurement models

The measurement models for the Burnout Scale (MBI-SSi), Engagement Scale (USEI), Social Support Scale (SS), Positive Coping Scale (BriefCOPE) and the Negative Coping Scale (BriefCOPE) presented a good fit to the data and all items displayed a distribution sufficiently close to normality. The loadings, residuals and reliability (Omega coefficient) of the indicators of the latent variables can be found in the S1 Appendix. The models’ fit to the data and maximum skewness and kurtosis for items in the different constructs are displayed in Table 2. Apart from Negative Coping and the Burnout*Engagement interaction were suboptimal omega coefficients were observed (ω = .61), all other constructs were measured with adequate factorial validity (CFI ≥ .95; TLI ≥.92; RMSEA ≤ .06; SRMR ≤ .04) and reliability (ω ≥ .8).

**Table 2:** Global measurement models’ fit (scaled indices), absolute maximum skewness and kurtosis for items in each construct and Omega L2 reliability.

Scale	df	χ2	CFI	TLI	RMSEA	SRMR	Max.|Ku|	Max.|Sk|	Omega L2
Burnout	78	756.4***	.973	.967	.046	.027	.89	1.40	.938
Engagement	40	356.5***	.975	.965	.044	.033	1.26	1.85	.817
Social Support	61	871.2***	.948	.922	.057	.042	1.03	.94	.911
Positive Coping	16	193.5***	.982	.969	.052	.033	.76	.74	.902
Negative Coping	19	73.9***	.994	.991	.027	.014	1.70	2.13	.613
Burnout*Engagement	15	50***	.982	.965	.038	.024	3.47	1.31	.612

(***p < .001).

Metric (weak) measurement invariance by country/region were observed according to the Rutkowski and Svetina [66] ΔCFI and ΔRMSEA criteria (see Table 3).

**Table 3:** Measurement models invariance by country/region.

Scale	ΔModel	ΔDf	Δχ 2	ΔCFI	ΔRMSEA
Burnout	Loadings-Configural	84	251.5***	-.004	-.002
	Intercepts-Loadings	84	1390.2***	-.031	.019
Engagement	Loadings-Configural	56	115.7***	-.004	-.001
	Intercepts-Loadings	56	661.17***	-.035	.020
Social Support	Loadings-Configural	70	526.9***	-.027	.006
	Intercepts-Loadings	70	1314.0***	-.062	.016
Positive Coping	Loadings-Configural	28	123.5***	-.008	.006
	Intercepts-Loadings	28	217.25***	-.013	.010
Negative Coping	Loadings-Configural	28	151.45***	-.015	.019
	Intercepts-Loadings	28	490.7***	-.033	.026
Burnout*Engagement	Loadings-Configural	84	251.2***	-.004	-.001
	Intercepts-Loadings	84	1390.2***	-.031	.019

(***p < .001).

In face of the weak measurement invariance by country, goodness of fit of the latent constructs were also assessed per country (S2 Appendix). The measurement models fit to the data from individual countries was acceptable for most constructs and participating countries/regions suggesting factorial validity (CFI ≥ .90; TLI ≥ .89 RMSEA ≤ .10; RMSEA ≤ .08) and reliability (ω ≥ .7). Exceptions to this overall goodness of fit were observed for Social Support in Serbia and Taiwan & Macao (ω = .107 and ω = .393 respectively). The Burnout vs. Engagement interaction construct also displayed sub-optimal reliability for most participants (ω ~.6; See S2 Appendix).

### 3.2. Student burnout mediation model

**Fig 2:**
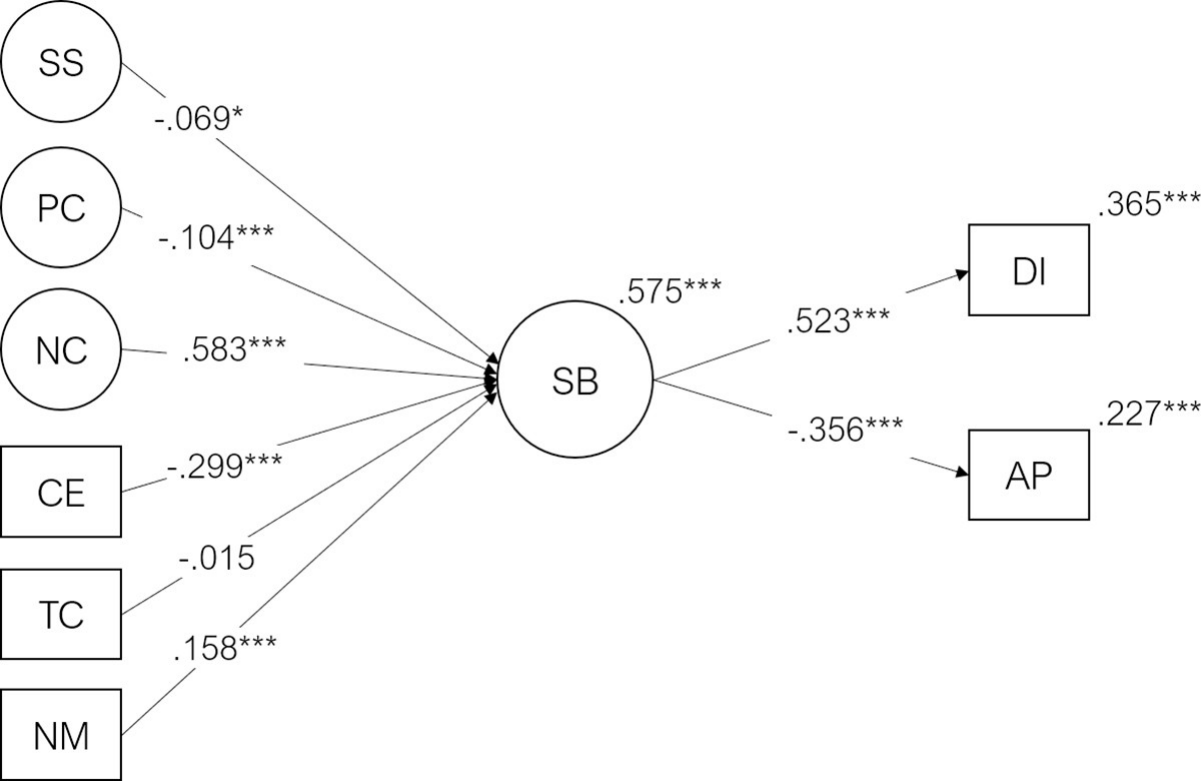
Student burnout mediation model. Standardized Regression coefficients (β), R^2^ and significance values for the burnout mediation model. CE = Course Expectations, TC = Teacher Competence, NM = Need for Medication, PC = Positive Coping, NC = Negative Coping, SS = Social Support, SB = Student Burnout, DI = Dropout Intention, AP = Academic Performance (***p < .001, **p < .01, *p≤.05, ^ns^ p>.05). For clarity, direct paths from the exogenous variables (SS, PC, NC, CE, TC, NM) to endogenous variables (DI, AP) were omitted. These standardized direct effects ranged from .012 (AP regressed on TC) to .119 (AP regressed on PC).

The model with six exogenous predictors (Course Expectations, Teacher Competence, Need for Medication, Social Support, Positive Coping, and Negative Coping), one mediator (Burnout) and two endogenous criterion variables (Academic Performance, and Dropout Intention) presented an acceptable fit to the data (χ^2^(1120) = 7512.0, p < .001, CFI = .925, TLI = .918, RMSEA = .037, SRMR = .064). The predictor variables explained 58% of the variability of the burnout factor (R^2^ = .575, p < .001). The burnout factor explained 37% of the variability of dropout intention (R^2^ = .365, p < .001) and 23% of the variability of academic performance (R^2^ = .226, p < .001). The standardized regression coefficients and p-values for this model are illustrated in Fig 2.

Negative Coping was the strongest predictor of student burnout (β = .583, p < .001), which, in turn, significantly predicted Dropout Intention (β = .523, p < .001) and Academic Performance (β = -.356, p < .001). On one hand, the standardized direct effects of Negative Coping over Dropout intention and Academic Performance was generally weak (β = .028 and β = -.091, respectively) and non-significant. On the other hand, the standardized indirect effects of Negative Coping, mediated by Burnout on Dropout Intention (β = .305, p < .001) and Academic Performance (β = -.208; p < .001) were medium-sized and statistically significant (p < .05).

### 3.3. Student engagement mediation model

**Fig 3:**
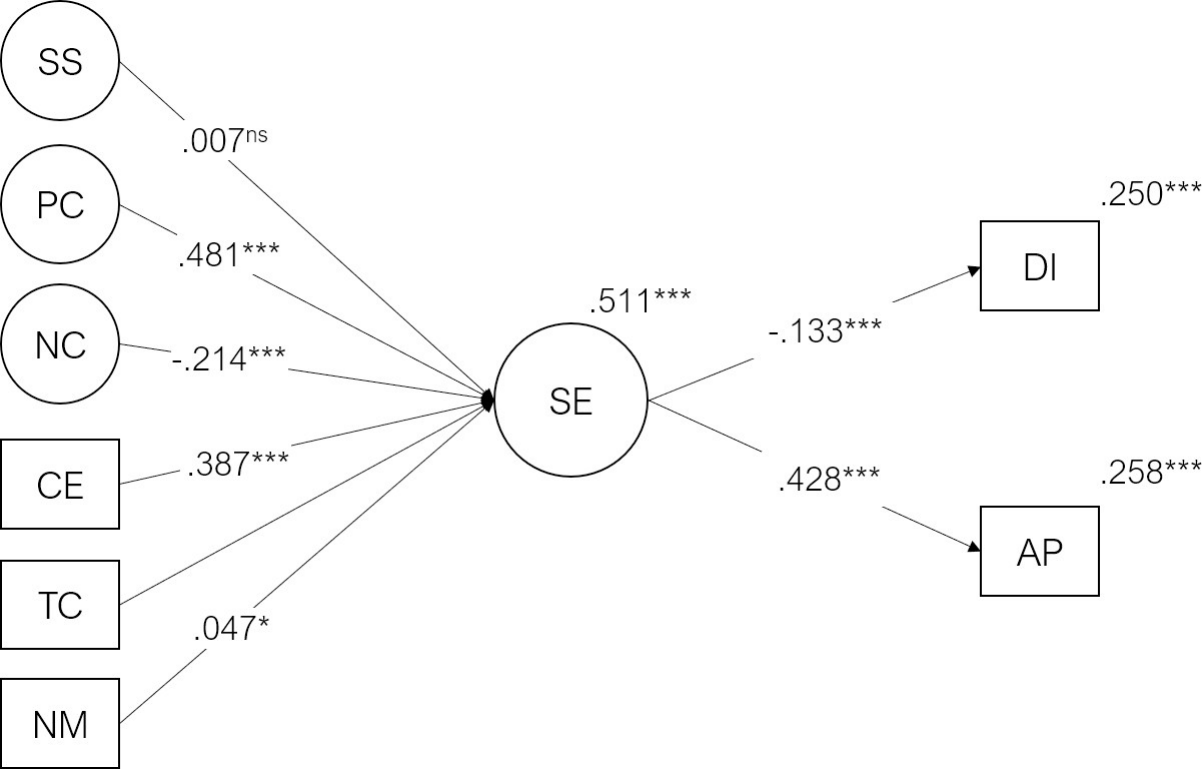
Student engagement mediation model. Standardized regression coefficients (β), significance and R^2^ values for the student engagement mediation model. CE = Course Expectations, TC = Teacher Competence, NM = Need for Medication, PC = Positive Coping, NC = Negative Coping, SS = Social Support, SE = Student Engagement, DI = Dropout Intention, AP = Academic Performance (***p < .001, **p < .01, *p≤.05, ^ns^ p>.05). For clarity, direct paths from the exogenous variables (SS, PC, NC, CE, TC, NM) to endogenous variables (DI and AP) were omitted. These standardized direct effects ranged from .000 to .286 (the path from Negative Coping to Dropout intention).

The model with six exogenous predictors (Course Expectations, Teacher Competence, Need for Medication, Social Support, Positive Coping. and Negative Coping), one mediator (Engagement) and two endogenous criterion variables (Academic Performance and Dropout Intention) presented an acceptable fit to the data (χ^2^(941) = 6347.7, p < .001, CFI = .914, TLI = .906, RMSEA = .038, SRMR = .060). The predictor variables explained 51% of the variability of engagement factor (R^2^ = .511, p < .001). This model (including the predictors’ direct and indirect effects) explained 25% of the variability of dropout intention (R^2^ = .25, p < .001), 26% of the variability of academic performance (R^2^ = .258, p < .001). The Standardized regression coefficients and p-values are shown in Fig 3.

Positive coping strategies and fulfillment of course expectations, were the strongest predictors of student engagement (β = .481, p < .001; β = .387, p < .001, respectively), which significantly predicted Academic Performance (β = .428, p < .001) and Dropout Intention (β = -.133, p < .001). The strongest standardized direct effect was the path from Negative Coping (β = .286, p < .001) and realization of Course Expectations (β = -.159; p < .001) to dropout intention. The strongest indirect effect, mediated by student engagement, on Academic Performance was observed for Positive Coping (β = .206; p < .001).

### 3.4. Burnout and engagement mediation model

**Fig 4:**
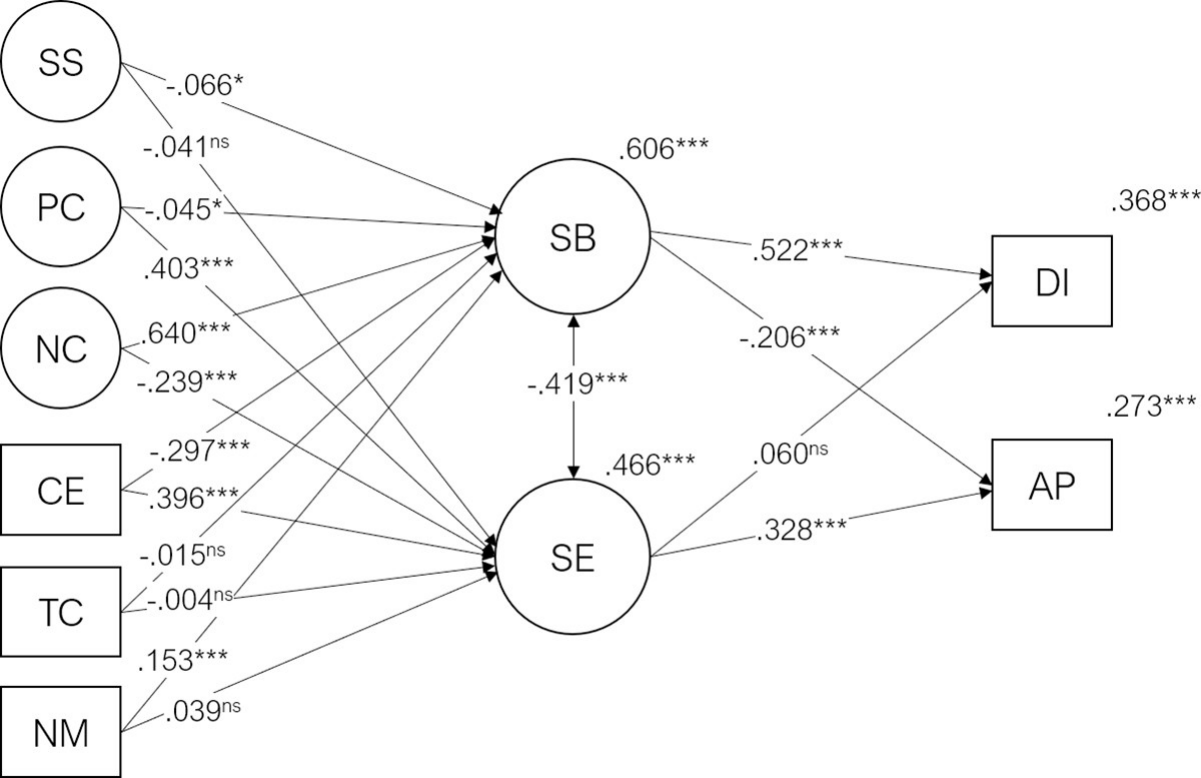
Student burnout and engagement mediation model. Standardized regression coefficients (β), significance and R^2^ values for the student engagement mediation model. CE = Course Expectations, TC = Teacher Competence, NM = Need for Medication, PC = Positive Coping, NC = Negative Coping, SS = Social Support, SE = Student Engagement, DI = Dropout Intention, AP = Academic Performance (***p < .001, **p < .01, *p≤.05, ^ns^ p>.05). For clarity, direct paths from the exogenous variables (SS, PC, NC, CE, TC, NM) to endogenous variables (DI, AP) were omitted. These standardized direct effects ranged from .016 to .075 (the path from Negative Coping to Dropout intention).

A conjoint model with Burnout and Engagement as mediators of Course Expectations, Teacher Competence, Need for Medication, Social Support, Positive Coping, and Negative Coping on Dropout Intention and Academic Performance showed an acceptable fit to the data (χ^2^(2189) = 11824.4, p < .001, CFI = .909, TLI = .903, RMSEA = .033, SRMR = .063). The predictor variables explained 61% of the variability of the burnout factor (R^2^ = .606, p < .001) and 47% of the variability of the engagement factor (R^2^ = .466, p < .001). As before, the strongest predictor of student burnout were negative coping strategies (β = .640; p < .001) and fulfillment of course expectations (β = -.297; p < .001), while for student engagement the strongest predictors were positive coping strategies and course expectations (β = .402; p < .001 and β = .398; p < .001 respectively). The mediation model of burnout and engagement (including the predictors’ direct and indirect effects) explained 37% of the variability of Dropout Intentions (R^2^ = .368, p < .001), and 27% of Academic Performance (R^2^ = .273, p < .001). As expected, there was a moderate negative correlation between Burnout and Engagement (r = -.419; p < .001). Fig 4 shows the standardized regression coefficients.

The strongest indirect effects were the ones between negative coping and dropout intention mediated by burnout (β = .334; p < .001) and positive coping strategies on subjective academic performance mediated by engagement (β = .132; p < .001). It is worthwhile to note that in the presence of the burnout construct the protective effect of student engagement over dropout intention is lost (compare Figs 3 and 4). The two constructs are significantly correlated and thus an interaction of engagement and burnout is both theoretically and empirically justifiable. This model is assessed next.

### 3.5. Burnout and engagement interaction model

A latent interaction between student burnout and engagement was added to the model described in Fig 4 to test for the possible interaction effects illustrated in Fig 1. This model showed an acceptable fit to the total data gathered in eight different countries and regions (χ^2^(2126) = 11723.1, p < .001, CFI = .910, TLI = .903, RMSEA = .033, SRMR = .062). The predictor variables explained 60% of the variability of the burnout factor (R^2^ = .601, p < .001) and 47% of the variability of the engagement (R^2^ = .466, p < .001). The model (including the predictors’ direct and indirect effects) explained 38% of the variability of the dropout intention (R^2^ = .378, p < .001) and 28% of the variability of academic performance (R^2^ = .278, p < .001). The regression coefficients and p-values for this model are provided in Fig 4.

**Fig 5:**
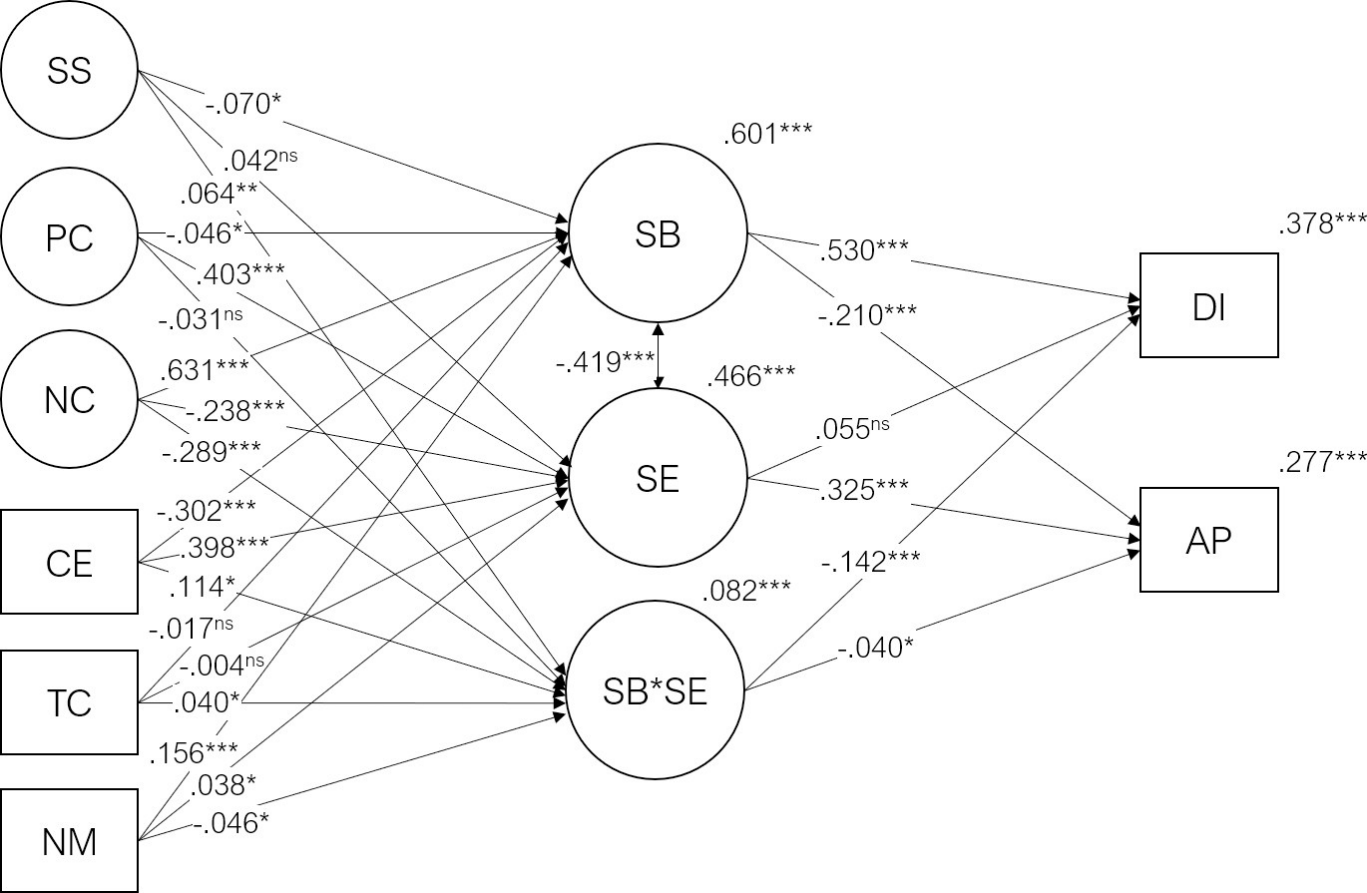
Burnout and engagement interaction model. Standardized regression coefficients (β), significance and R^2^ values for the student burnout and engagement interaction model. CE = Course Expectations, TC = Teacher Competence, NM = Need for Medication, PC = Positive Coping, NC = Negative Coping, SS = Social Support, SB = Student Burnout, SE = Student Engagement, SB*SE = Student Burnout, and Student Engagement Interaction, DI = Dropout Intention, AP = Academic Performance (***p < .001, **p < .01, *p≤.05, ^ns^ p>.05). For clarity, direct paths from the exogenous variables (SS, PC, NC, CE, TC, NM) to endogenous variables (DI, AP) were omitted. Standardized direct effects ranged from -.001 to -.106 (AP regressed on NC).

The strongest burnout and engagement interaction effect was observed on the dropout intention (β = -.142; p < .001). The negative sign of the interaction means that when student burnout increases, the effect of student engagement on dropout intention is attenuated (or vice-versa) and, in the presence of high burnout, the protective effect of engagement is suppressed when predicting dropout (β = .055; p = .086). To test this hypothesis and quantify the size of this suppression effect, the regressions weights associated with engagement from model in Fig 3 [Dropout Intention = *a*×Engagement + (…)] and Fig 5 [Dropout Intention = Burnout + *b*×Engagement + Burnout*Engagement + (…)] were evaluated. The estimated suppression effects (*a* − *b*) were tested with a Z-test for large samples. The suppression effect was significantly different from 0 when predicting students dropout intentions (|*a*–*b*| = 0.188, p < .001) and academic performance (|*a*–*b*| = 0.103, p = .044). The interaction effect of student burnout and student engagement also had a negative but relatively small impact on academic performance (β = -.04, p = .036) suggesting that the effect of Burnout on self-reported academic performance was reduced when the Engagement is high (and vice-versa).

### 3.6. Invariance of the burnout and engagement interaction model

Using the Cheung and Rensvold (2002) ΔCFI criterion [65] and the Rutkowski and Svetina (2014) ΔRMSEA criterion [66], strict invariance of the Burnout and Engagement Interaction Model was found between genders and areas of study (Table 4). However, only weak country/region invariance was observed (Table 4), once only configural and metric invariance was observed. Since the equality of intercepts assumption was not met, no further invariance analysis was pursued [58, 63]. The Burnout and Engagement interaction model was thereafter individually fitted for each participating country/region.

**Table 4:** Gender, area of study and country/regions analysis of invariance for the SE*SB interaction model.

Model	df	χ^2^	CFI	TLI	RMSEA	SRMR	Δdf	Δχ^2^	ΔCFI	ΔRMSEA
Gender										
Configural	4244	16881	.905	.897	.037	.064				
Loadings	4288	16987	.905	.898	.037	.064	44	106***	.000	.000
Intercepts	4332	17388	.905	.896	.038	.064	44	401***	.000	.000
Means	4351	17431	.902	.896	.037	.064	19	43**	-.003	.000
Regressions	4364	17630	.900	.895	.038	.064	13	199***	-.002	.001
Structural	4400	17682	.900	.896	.038	.064	45	52***	.000	.000
Area of Study										
Configural	8488	22465	.897	.888	.039	.066				
Loadings	8620	22751	.896	.889	.039	.067	132	286***	-.001	.000
Intercepts	8752	23256	.893	.888	.039	.067	132	505***	-.003	.000
Means	8809	23413	.892	.887	.039	.068	57	157***	-.001	.000
Regressions	8848	23683	.891	.886	.039	.068	39	270***	-.001	.000
Structural	8956	23889	.890	.887	.039	.068	108	206***	-.002	.000
County/region										
Configural	16976	35141	.874	.865	.044	.073				
Loadings	17284	36776	.865	.858	.045	.078	308	1635***	-.009	.001
Intercepts	17592	41127	.835	.829	.050	.081	308	4351***	-.030	.005

### 3.7. Student burnout and engagement interaction model per country/region

Since only weak (metric) measurement invariance was found for the overall Burnout and Engagement interaction model per country/region, this interaction model was fitted to the samples from each of the countries/regions separately. Configural invariance (that is the same model configuration between countries/regions) was found previously (see Table 4) suggesting that all variables in the model presented in Fig 5 may play a similar role for the different countries/regions. On the other hand, metric but not scalar invariance (equal loadings but different intercepts for the measurement model) was found assuring the validity of structural weights comparisons between countries [58, 63]. Table 5 resumes the standardized structural paths for each variable in the model, variability of dropout intention and academic performance (R^2^) and model fit indices.

**Table 5:** Standardized structural paths, R^2^ and fit indices for the SE*SB interaction model per country/region.

Sample	Standardized Structural Paths											R^2^	Fit Indices
Portugal	PC	−	-.014	^ns^	→	SB							
	NC	−	.659	***	→								
	SS	−	-.111	^ns^	→		−	.426	***	→	DI		
	CE	−	-.278	***	→		−	-.120	^ns^	→	AP		
	TC	−	-.014	^ns^	→								
	NM	−	.098	***	→								
	PC	−	.398	***	→	SE							
	NC	−	-.256	**	→								
	SS	−	.036	^ns^	→		−	-.021	^ns^	→	DI		
	CE	−	.363	***	→		−	.420	***	→	AP		
	TC	−	.010	^ns^	→								
	NM	−	.017	^ns^	→								
	PC	−	-.052	^ns^	→	SB*SE							
	NC	−	-.210	*	→								CFI = .889
	SS	−	-.026	^ns^	→		−	-.127	**	→	DI	.394	TLI = .881
	CE	−	.164	^ns^	→		−	-.014	^ns^	→	AP	.307	RMSEA = .039
	TC	−	.056	^ns^	→								SRMR = .072
	NM	−	-.050	^ns^	→								χ^2^/df = 2.6
	PC	−	.016	^ns^	→	DI							
	NC	−	.092	^ns^	→								
	SS	−	-.041	^ns^	→								
	CE	−	-.124	***	→								
	TC	−	-.013	^ns^	→								
	NM	−	.088	**	→								
	PC	−	-.030	^ns^	→	AP							
	NC	−	-.167	*	→								
	SS	−	-.161	^ns^	→								
	CE	−	.026	^ns^	→								
	TC	−	.034	^ns^	→								
	NM	−	.008	^ns^	→					^ ^			
Brazil	PC	−	.086	^ns^	→	SB							
	NC	−	.681	***	→								
	SS	−	-.172	*	→		−	.622	***	→	DI		
	CE	−	-.308	***	→		−	.012	^ns^	→	AP		
	TC	−	.005	^ns^	→								
	NM	−	.120	**	→								
	PC	−	.156	^ns^	→	SE							
	NC	−	-.368	*	→								
	SS	−	.120	^ns^	→		−	-.017	^ns^	→	DI		
	CE	−	.508	***	→		−	.403	**	→	AP		
	TC	−	.034	^ns^	→								
	NM	−	.029	^ns^	→								
	PC	−	-.076	^ns^	→	SB*SE							
	NC	−	-.450	*	→								CFI = .877
	SS	−	.007	^ns^	→		−	-.200	**	→	DI	.477	TLI = .868
	CE	−	.301	^ns^	→		−	-.111	*	→	AP	.349	RMSEA = .043
	TC	−	.051	^ns^	→								SRMR = .074
	NM	−	.067	^ns^	→								χ^2^/df = 1.8
	PC	−	.029	^ns^	→	DI							
	NC	−	-.112	^ns^	→								
	SS	−	-.082	^ns^	→								
	CE	−	.012	^ns^	→								
	TC	−	.010	^ns^	→								
	NM	−	.032	^ns^	→								
	PC	−	-.109	^ns^	→	AP							
	NC	−	-.430	*	→								
	SS	−	-.072	^ns^	→								
	CE	−	-.015	^ns^	→								
	TC	−	.045	^ns^	→								
	NM	−	-.035	^ns^	→					^ ^			
Mozambique	PC	−	-.352	*	→	SB							
	NC	−	.856	*	→								
	SS	−	-.057	^ns^	→		−	.317	*	→	DI		
	CE	−	-.248	***	→		−	-.853	***	→	AP		
	TC	−	.087	^ns^	→								
	NM	−	.082	^ns^	→								
	PC	−	.732	***	→	SE							
	NC	−	-.405	*	→								
	SS	−	.078	^ns^	→		−	-.056	^ns^	→	DI		
	CE	−	.259	***	→		−	.325	**	→	AP		
	TC	−	.046	^ns^	→								
	NM	−	.005	^ns^	→								
	PC	−	-.484	*	→	SB*SE							
	NC	−	.776	**	→								CFI = .815
	SS	−	-.003	^ns^	→		−	-.114	*	→	DI	.220	TLI = .802
	CE	−	-.196	*	→		−	.017	^ns^	→	AP	.339	RMSEA = .038
	TC	−	.012	^ns^	→								SRMR = .065
	NM	−	.011	^ns^	→								χ^2^/df = 1.6
	PC	−	-.027	^ns^	→	DI							
	NC	−	.089	^ns^	→								
	SS	−	-.033	^ns^	→								
	CE	−	-.220	*	→								
	TC	−	.040	^ns^	→								
	NM	−	.044	^ns^	→								
	PC	−	.391	*	→	AP							
	NC	−	-.642	*	→								
	SS	−	.086	^ns^	→								
	CE	−	-.049	^ns^	→								
	TC	−	-.010	^ns^	→								
	NM	−	.085	^ns^	→					^ ^			
UK	PC	−	-.016	^ns^	→	SB							
	NC	−	.404	***	→								
	SS	−	-.277	^ns^	→		−	.859	***	→	DI		
	CE	−	-.443	***	→		−	-.563	**	→	AP		
	TC	−	.070	^ns^	→								
	NM	−	.077	^ns^	→								
	PC	−	.419	**	→	SE							
	NC	−	-.106	^ns^	→								
	SS	−	.256	*	→		−	.353	**	→	DI		
	CE	−	.381	***	→		−	.048	^ns^	→	AP		
	TC	−	.052	^ns^	→								
	NM	−	.015	^ns^	→								
	PC	−	-.027	^ns^	→	SB*SE							
	NC	−	-.164	^ns^	→								CFI = .874
	SS	−	.054	^ns^	→		−	-.191	*	→	DI	.419	TLI = .865
	CE	−	.130	^ns^	→		−	.033	^ns^	→	AP	.372	RMSEA = .043
	TC	−	-.028	^ns^	→								SRMR = .079
	NM	−	.099	^ns^	→								χ^2^/df = 1.6
	PC	−	-.189	^ns^	→	DI							
	NC	−	-.134	^ns^	→								
	SS	−	-.010	^ns^	→								
	CE	−	-.004	^ns^	→								
	TC	−	-.109	^ns^	→								
	NM	−	.002	^ns^	→								
	PC	−	.259	*	→	AP							
	NC	−	.082	^ns^	→								
	SS	−	-.101	^ns^	→								
	CE	−	-.008	^ns^	→								
	TC	−	.101	*	→								
	NM	−	-.002	^ns^	→					^ ^			
USA	PC	−	-.059	^ns^	→	SB							
	NC	−	.772	***	→								
	SS	−	.077	^ns^	→		−	.023	^ns^	→	DI		
	CE	−	-.441	***	→		−	-.197	**	→	AP		
	TC	−	-.028	^ns^	→								
	NM	−	.098	*	→								
	PC	−	.325	*	→	SE							
	NC	−	-.419	**	→								
	SS	−	-.049	^ns^	→		−	.018	^ns^	→	DI		
	CE	−	.343	***	→		−	.379	**	→	AP		
	TC	−	.115	^ns^	→								
	NM	−	.073	^ns^	→								
	PC	−	.084	^ns^	→	SB*SE							
	NC	−	-.366	^ns^	→								CFI = .893
	SS	−	-.160	^ns^	→		−	-.198	*	→	DI	.342	TLI = .886
	CE	−	.258	*	→		−	-.085	^ns^	→	AP	.332	RMSEA = .041
	TC	−	.028	^ns^	→								SRMR = .073
	NM	−	.045	^ns^	→								χ^2^/df = 1.5
	PC	−	-.073	^ns^	→	DI							
	NC	−	.420	**	→								
	SS	−	-.012	^ns^	→								
	CE	−	-.099	^ns^	→								
	TC	−	.096	^ns^	→								
	NM	−	.107	^ns^	→								
	PC	−	-.041	^ns^	→	AP							
	NC	−	-.116	^ns^	→								
	SS	−	-.102	^ns^	→								
	CE	−	.062	^ns^	→								
	TC	−	-.042	^ns^	→								
	NM	−	-.051	^ns^	→					^ ^			
Finland	PC	−	-.115	^ns^	→	SB							
	NC	−	.678	***	→								
	SS	−	.074	^ns^	→		−	.131	*	→	DI		
	CE	−	-.387	***	→		−	-.235	**	→	AP		
	TC	−	.006	^ns^	→								
	NM	−	.137	*	→								
	PC	−	.549	**	→	SE							
	NC	−	-.123	^ns^	→								
	SS	−	.102	^ns^	→		−	-.258	**	→	DI		
	CE	−	.379	***	→		−	.336	***	→	AP		
	TC	−	-.038	^ns^	→								
	NM	−	-.085	^ns^	→								
	PC	−	.002	^ns^	→	SB*SE							
	NC	−	-.368	*	→								CFI = .863
	SS	−	-.076	^ns^	→		−	-.092	^ns^	→	DI	.380	TLI = .853
	CE	−	.084	^ns^	→		−	-.063	^ns^	→	AP	.290	RMSEA = .046
	TC	−	-.044	^ns^	→								SRMR = .088
	NM	−	.051	^ns^	→								χ^2^/df = 1.8
	PC	−	.007	^ns^	→	DI							
	NC	−	.236	^ns^	→								
	SS	−	.079	^ns^	→								
	CE	−	-.207	**	→								
	TC	−	-.027	^ns^	→								
	NM	−	.106	^ns^	→								
	PC	−	-.042	^ns^	→	AP							
	NC	−	-.192	*	→								
	SS	−	-.174	^ns^	→								
	CE	−	-.002	^ns^	→								
	TC	−	.020	^ns^	→								
	NM	−	-.039	^ns^	→					^ ^			
Serbia	PC	−	-.047	^ns^	→	SB							
	NC	−	.401	**	→								
	SS	−	.062	^ns^	→		−	.485	***	→	DI		
	CE	−	-.435	***	→		−	-.218	*	→	AP		
	TC	−	-.119	*	→								
	NM	−	.245	***	→								
	PC	−	.264	*	→	SE							
	NC	−	-.216	^ns^	→								
	SS	−	-.023	^ns^	→		−	.119	*	→	DI		
	CE	−	.389	***	→		−	.097	^ns^	→	AP		
	TC	−	.108	^ns^	→								
	NM	−	-.079	^ns^	→								
	PC	−	-.063	^ns^	→	SB*SE							
	NC	−	-.344	*	→								CFI = .839
	SS	−	-.089	^ns^	→		−	-.149	*	→	DI	.357	TLI = .828
	CE	−	.149	^ns^	→		−	.047	^ns^	→	AP	.326	RMSEA = .050
	TC	−	.025	^ns^	→								SRMR = .089
	NM	−	.051	^ns^	→								χ^2^/df = 1.8
	PC	−	.018	^ns^	→	DI							
	NC	−	.169	^ns^	→								
	SS	−	.050	^ns^	→								
	CE	−	-.117	^ns^	→								
	TC	−	-.031	^ns^	→								
	NM	−	-.041	^ns^	→								
	PC	−	.104	^ns^	→	AP							
	NC	−	-.117	^ns^	→								
	SS	−	.032	^ns^	→								
	CE	−	.256	***	→								
	TC	−	-.146	**	→								
	NM	−	-.026	^ns^	→					^ ^			
Taiwan & Macao	PC	−	-.047	^ns^	→	SB							
	NC	−	1.248	***	→								
	SS	−	.484	^ns^	→		−	.524	***	→	DI		
	CE	−	-.240	***	→		−	-.341	**	→	AP		
	TC	−	.018	^ns^	→								
	NM	−	.007	^ns^	→								
	PC	−	.669	***	→	SE							
	NC	−	.111	^ns^	→								CFI = .881
	SS	−	.245	*	→		−	.085	^ns^	→	DI	.252	TLI = .873
	CE	−	.233	***	→		−	.277	*	→	AP	.331	RMSEA = .040
	TC	−	-.039	^ns^	→								SRMR = .073
	NM	−	-.014	^ns^	→								χ^2^/df = 2.2
	PC	−	-.154	^ns^	→	SB*SE							
	NC	−	-.838	**	→								
	SS	−	-.554	^ns^	→		−	-.097	^ns^	→	DI		
	CE	−	.022	^ns^	→		−	-.030	^ns^	→	AP		
	TC	−	.004	^ns^	→								
	NM	−	-.113	^ns^	→								
	PC	−	-.061	^ns^	→	DI							
	NC	−	-.152	^ns^	→								
	SS	−	-.153	^ns^	→								
	CE	−	-.029	^ns^	→								
	TC	−	-.032	^ns^	→								
	NM	−	.075	^ns^	→								
	PC	−	.070	^ns^	→	AP							
	NC	−	.079	^ns^	→								
	SS	−	-.006	^ns^	→								
	CE	−	.126	*	→								
	TC	−	-.010	^ns^	→								
	NM	−	-.033	^ns^	→					^ ^			

CE = Course Expectations, TC = Teacher Competence, NM = Need for Medication, PC = Positive Coping, NC = Negative Coping, SS = Social Support, SB = Student Burnout, SE = Student Engagement, SB*SE = Student Burnout and Student Engagement Interaction, DI = Dropout Intention, AP = Academic Performance (***p < .001, **p < .01, *p≤.1, ^ns^ p>.1).

Overall model fit for individual countries/regions were acceptable (although CFI and TLI had lower than acceptable values, RMSEA, SRMR and χ^2^/df were indicative of very good fit). Variance of dropout intention explained by the model ranged from 22% (in Mozambique) to 48% (in Brazil). Explained variability for subjective academic performance ranged from 29% (in Finland) to 37% (in the UK).

Negative coping strategies were the strongest and most statistically significant predictors of student burnout (standardized beta weights ranging from around .4-.7 in the United Kingdom, Portugal, Brazil, Finland, Serbia, to .8-.9 in the USA, and Taiwan & Macao). For Student Engagement, no consistent predictors were found among countries/regions. Positive coping strategies were strongest predictors of Engagement in Portugal, the United Kingdom, Finland, and Mozambique with standardized effects around .5 to .7, followed closely by the fulfillment of course expectations with standardized effects of .2-.4 (see Table 5). This variable was the strongest positive predictor of the United Kingdom and the USA students’ engagement (β = .30). Overall, positive coping strategies and fulfillment of course expectations, accounted for most of the explained variance of student engagement among countries/regions participating in this study. Further, negative coping and non-fulfillment of course expectations were the strongest predictors for student burnout. Student burnout was the most consistent predictor of dropout intention for all countries/regions with effect sizes (standardized beta) ranging from .3 for Mozambique to .9 in the United Kingdom. Surprisingly, the effect of student burnout on dropout intention was not significant in the USA, after accounting for the direct effect of negative coping strategies on drop out intention (β = .42) and the correlation between burnout and engagement (r = -.51). Student engagement *per se* had no significant effect for most participants but displayed a protective effect (negative interaction) over the student burnout effect on intention to dropout. Student engagement strongest effects were observed on the self-related academic performance with average standardized effects between .2 and .4 for most participants.

## 4 Discussion

The academic and social demands of university life exert pressure on students’ cognitive and emotional resources, often leading to exhaustion. This exhaustion, combined with self-inefficacy, and cynicism towards studies form the Burnout Syndrome triad. Burnout has been identified as one of the leading causes of poor student performance and intention of dropout [17, 24]. Conversely, student engagement is seen as a protective variable that prevents the aggravating effects of burnout on students’ well-being [9]. In this study, we analyze some of the causes and consequences of student engagement and burnout. Social support, coping strategies, teacher competency (as rated by students), fulfillment course expectations and need for medication were used as predictors of student engagement and burnout, which in turn were used as predictors of subjective academic performance, and dropout intention.

We found that negative coping strategies (such as disengagement, self-distraction, denial, self-blame and substance abuse) were the strongest predictors of student burnout among most countries/regions, a result that follow previous findings [32, 35]. Student burnout, in turn, was highly predictive of students’ dropout intention, and poor academic performance, a result that has been also observed in other studies [17, 24]. Its effects are however not consistent in all countries/regions. Strongest effects were observed in western participants (e.g. Portugal and the UK) with the lowest effects observed in African (Mozambique) and North Europe (Finland) participants. These differences may be attributable to cultural factors and differential valuing of higher education and coping mechanisms among western countries versus other regions. The greatest predictors of student engagement were positive coping strategies, positive course expectations, and positive perceptions of teacher competence, with standardized effect consistent among participants. Student engagement, in turn, was a good predictor of subjective academic performance and dropout intention, a result that has been observed in previous studies [67]. Except for the prediction of student engagement by the need for medication and social support, all hypothesized causes (H1 and H2) and consequences (H3 and H4) of student engagement and burnout were statistically significant, and the models representing the relationships among these variables presented an acceptable/good fit to the data, thus confirming our hypotheses H1 to H4.

Because the burnout syndrome has substantial overlap with depression symptoms [68], the need for medication was a significant, but weak, predictor of student burnout in most country/regions, but not of student engagement. However, highly engaged students may use medication for cognitive enhancement purposes [69], therefore nullifying the expected negative relationship between these variables. By specifying the type and purpose of the medication students need, stronger correlations with student engagement and burnout might be observed.

Social support was not a predictor of student engagement in most participants, with the UK being the only exception, a result that was not in line with previous findings [70]. One possible interpretation of this result is that, due to covariation, the social support effect loses importance when considered simultaneously with other factors (e.g. coping strategies).

After performing the analysis of separate models of student burnout and student engagement, we tested the interaction effect of student engagement and burnout on dropout intention and self-reported academic performance. We found that student engagement and burnout show a significant interaction effect, albeit with small effect size, when explaining dropout intention but not academic performance. The data suggest that student engagement can attenuate the effect of burnout on dropout intention, validating our fifth hypothesis (H5). However, this effect was not consistent in all countries/regions. The constructs used in the engagement/burnout interaction model and their relationships were invariant across gender and different areas of this study. However, the model was not fully invariant across countries, partially validating our fourth hypothesis (H6). This result suggests a need for local specific analysis considering the cultural and societal contexts and the different cultures’ valuing of higher education.

It is possible that the statistical significance of small observed effect of student burnout interaction with engagement on the criterion variables was due to the overall large sample size used in this study and its associated high level of statistical power. To the best of our knowledge, there are no other studies that have probed the interaction effect of student engagement and student burnout on these outcomes. Thus, further replication is necessary before a moderation effect of the two constructs can be introduced in models examining the effects of burnout and engagement on student’s academic life and well-being. Our models do show that in the presence of burnout, the effect of student engagement on dropout intentions and academic performance is attenuated. That is, engagement in one’s studies may not have expected effects on academic performance and degree completion if high levels of burnout related to studies develop. The first interpretation of this result is that when student engagement is associated with perfectionism, this can lead to burnout which in turn can affect academic performance, an interpretation supported by recent research [35]. The second interpretation is that it may have occurred merely because of a suppression effect due to the relationship between student engagement and burnout. A suppression effect can happen in multiple structural equation models when two or more predictor variables are strongly associated [71, 72]. For instance, after removing the common variance between student burnout and engagement, which are negatively correlated, student engagement loses its predictive power over dropout intention. This suppression effect may result from the fact that, when we take into consideration the prevalence of burnout in university students, engagement levels become less relevant when predicting important academic outcomes.

The results of this study have strong implications for practitioners and administrators. Data gathered from a large sample of university students from eight countries and regions around the world show that, while promoting student engagement is important to prevent dropout, major efforts (e.g., reducing course-work load or increased coordination between teachers teaching different subjects) must be pursued to prevent student burnout. Student engagement alone cannot prevent dropout intentions and promote good academic performance if student burnout is high. Our results suggest that culture features and school valuing may play a significant role in the comprehension of burnout syndrome and its relationship with other relevant variables. Therefore, it would be important to conceptualize future interventions taking into account specific cultural contexts.

## 5 Limitations

Because of the cross-sectional nature of the current study causation inferred from the data must be cautioned. Causation implies a correlation, but the reverse may not necessarily be true. It is important to avoid causal interpretations of the results, as that would require that longitudinal and experimental methods be used. Therefore, although causal relationships between these variables are implied by structural equation models, caution needs to be taken concerning the causal nature of their relationships. This is a strong limitation common to correlational studies.

A second limitation of this study is the self-reported nature of the data collected, namely on the criterion variables, which may create a negativity bias. However, “in reference to variables such as burnout and engagement, self-reports may be the most accurate form of assessment, as the individual is the best person to report one’s own affective state.” [32, p. 224]. Also, given that data collection was carried out via an online survey, a self-selection bias also may be present. Unfortunately, most psychology studies will suffer from a self-selection bias as participants with certain characteristics may refuse to participate in such studies.

Finally, a third limitation regards the poor fit of some measurement models in some of the countries/regions. Face value of higher education, multicultural, educational, and social diversity and norms in such diverse countries as Portugal, Finland, Serbia, Mozambique, The United States of America or Taiwan & Macao may hinder the generalizability of a full model of student burnout and engagement effects on college dropout and academic efficacy, even that a set of consistent predictors emerged from this study.

## 6 Conclusions

Coping strategies, course expectations, need for medication and social support significantly predicted student burnout. Student engagement was significantly predicted by coping strategies and course expectations. Both student engagement and student burnout significantly predicted subjective academic performance, but only Burnout predicted dropout intentions. Negative coping was the strongest predictor of burnout which in turn was the strongest predictor of dropout intention. Student engagement was predicted by positive coping strategies, as well as course expectations, to a lesser extent by negative coping strategies, but not social support.

Most surprisingly, burnout levels suppressed the effect of student engagement on dropout intention when an interaction between the two constructs is present. A negative and significant interaction of burnout and engagement on dropout intentions revealed that effect of burnout on dropout is attenuated by engagement. Given that this study solicited the participation of over four thousand participants from different countries/regions and areas of study, we believe that this is a noteworthy result that has important implications for educational psychologists, practitioners, and administrators.

To prevent student dropout and promote academic performance it is not enough to promote student engagement—student burnout also must be kept as low as possible. Other variables such as coping strategies and social support also are relevant predictors of student engagement and burnout and should be considered when implementing preventive actions, self-help, and guided intervention programs for college students.

## Supporting information

S1 Appendix Loadings and residuals of the indicators of the latent variables. (DOCX)

S2 Appendix Measurement models’ fit by country. (DOCX)
