# Supplementary material for: Predictors of academic efficacy and dropout intention in university students: Can engagement suppress burnout?
Source: PLoS One. 2020 Oct 29;15(10):e0239816. doi: 10.1371/journal.pone.0239816 (PMC7595383; doi:10.1371/journal.pone.0239816)
Supplement: S1 Appendix — (DOCX) [file pone.0239816.s001.docx]

**S1 Appendix: Loading, residuals and reliability of the indicators of the latent variables.**

| Latent Factor | Factor/Indicator | Loading | Residual | OmegaL2/Omega |
| --- | --- | --- | --- | --- |
| Burnout | Exhaustion | .807 | .349 | .938 |
|  | Cynicism | .945 | .107 |  |
|  | Inefficacy | .926 | .143 |  |
| Exhaustion | MBI1 | .802 | .357 | .882 |
|  | MBI2 | .716 | .487 |  |
|  | MBI3 | .753 | .433 |  |
|  | MBI4 | .825 | .319 |  |
|  | MBI5 | .864 | .254 |  |
| Cynicism | MBI6 | .804 | .354 | .815 |
|  | MBI7 | .826 | .318 |  |
|  | MBI8 | .816 | .334 |  |
|  | MBI9 | .704 | .504 |  |
| Inefficacy | MBI10 | .789 | .377 | .854 |
|  | MBI11 | .726 | .473 |  |
|  | MBI12 | .745 | .445 |  |
|  | MBI13 | .668 | .554 |  |
|  | MBI14 | .630 | .603 |  |
|  | MBI15 | .786 | .382 |  |
| Engagement | Behavioral Engagement | .880 | .226 | .825 |
|  | Emotional Engagement | .736 | .458 |  |
|  | Cognitive Engagement | .634 | .598 |  |
| Behavioral Engagement | SE1 | .608 | .630 | .674 |
|  | SE4 | .642 | .588 |  |
|  | SE5 | .623 | .612 |  |
| Emotional Engagement | SE7 | .742 | .449 | .859 |
|  | SE8 | .799 | .362 |  |
|  | SE9 | .877 | .231 |  |
|  | SE10 | .687 | .528 |  |
| Cognitive Engagement | SE12 | .540 | .708 | .781 |
|  | SE13 | .594 | .647 |  |
|  | SE14 | .835 | .303 |  |
|  | SE15 | .770 | .407 |  |
| Social Support | Satisfaction with Friends | .853 | .272 | .911 |
|  | Intimacy | .963 | .073 |  |
|  | Social Activities | .508 | .742 |  |
|  | Satisfaction with Family | .520 | .730 |  |
| Satisfaction with Friends | SS1 | .598 | .642 | .825 |
|  | SS2 | .736 | .458 |  |
|  | SS3 | .655 | .571 |  |
|  | SS4 | .706 | .502 |  |
|  | SS5 | .729 | .469 |  |
| Intimacy | SS6 | .421 | .823 | .554 |
|  | SS7 | .650 | .578 |  |
|  | SS8 | .668 | .554 |  |
| Social Activities | SS10 | .825 | .319 | .707 |
|  | SS11 | .482 | .768 |  |
|  | SS12 | .727 | .471 |  |
| Satisfaction with Family | SS13 | .727 | .471 | .753 |
|  | SS14 | .884 | .219 |  |
|  | SS15 | .436 | .810 |  |
| Positive Coping | Active Coping | .945 | .107 | .902 |
|  | Planning | .892 | .204 |  |
|  | Instrumental Support | .541 | .707 |  |
|  | Positive Reframing | .508 | .742 |  |
| Active Coping | BC1 | .732 | .464 | .785 |
|  | BC2 | .872 | .240 |  |
| Planning | BC3 | .897 | .195 | .788 |
|  | BC4 | .711 | .494 |  |
| Instrumental Support | BC5 | .871 | .241 | .785 |
|  | BC6 | .730 | .467 |  |
| Positive Reframing | BC11 | .870 | .243 | .848 |
|  | BC12 | .847 | .283 |  |
| Negative Coping | Self Blame | .507 | .743 | .613 |
|  | Denial | .322 | .896 |  |
|  | Behavioral Disengagement | .767 | .412 |  |
|  | Substance Use | .367 | .865 |  |
| Self Blame | BC13 | .614 | .623 | .802 |
|  | BC14 | .963 | .073 |  |
| Denial | BC19 | .729 | .469 | .831 |
|  | BC20 | .956 | .086 |  |
| Behavioral Disengagement | BC23 | .877 | .231 | .874 |
|  | BC24 | .885 | .217 |  |
| Substance Use | BC25 | .956 | .086 | .996 |
|  | BC26 | .949 | .099 |  |
| Burnout*Engagement | SE4.MBI4 | .302 | .909 |  |
|  | SE5.MBI5 | .288 | .917 |  |
|  | SE6.MBI6 | .495 | .755 |  |
|  | SE7.MBI7 | .704 | .504 | .612 |
|  | SE8.MBI8 | .531 | .718 |  |
|  | SE9.MBI9 | .554 | .693 |  |
|  | SE15.MBI15 | .270 | .927 |  |

* All loadings are statistically significant for *p* <.001
