# Supplementary material for: Predictors of academic efficacy and dropout intention in university students: Can engagement suppress burnout?
Source: PLoS One. 2020 Oct 29;15(10):e0239816. doi: 10.1371/journal.pone.0239816 (PMC7595383; doi:10.1371/journal.pone.0239816)
Supplement: S2 Appendix — (DOCX) [file pone.0239816.s002.docx]

**S2 Appendix: Measurement models’ fit (scaled indices) and reliability (omega L2 or omega) by country.**

| Sample | Construct | df | χ2 | CFI | TLI | RMSEA | SRMR | Omega |
| --- | --- | --- | --- | --- | --- | --- | --- | --- |
| Portugal | Burnout | 78 | 376.974*** | .964 | .951 | .060 | .033 | .924 |
|  | Engagement | 40 | 163.25*** | .966 | .953 | .054 | .039 | .785 |
|  | Social Support | 61 | 413.292*** | .934 | .901 | .074 | .058 | .829 |
|  | Positive Coping | 16 | 51.478*** | .987 | .977 | .046 | .032 | .775 |
|  | Negative Coping | 19 | 37.367** | .992 | .988 | .030 | .026 | .630 |
|  | Burnout*Engagement | 10 | 29.671*** | .963 | .922 | .043 | .032 | .673 |
| Brazil | Burnout | 78 | 209.006*** | .960 | .946 | .063 | .035 | .931 |
|  | Engagement | 40 | 82.754*** | .972 | .961 | .050 | .042 | .826 |
|  | Social Support | 61 | 163.295*** | .941 | .911 | .063 | .056 | .818 |
|  | Positive Coping | 16 | 31.598** | .985 | .974 | .048 | .049 | .717 |
|  | Negative Coping | 19 | 25.422^ns^ | .995 | .992 | .028 | .031 | .618 |
|  | Burnout*Engagement | 10 | 13.695* | .991 | .981 | .030 | .034 | .749 |
| Mozambique | Burnout | 78 | 134.41*** | .965 | .952 | .042 | .037 | .910 |
|  | Engagement | 40 | 39.665^ns^ | 1.000 | 1.001 | .000 | .028 | .813 |
|  | Social Support | 61 | 131.739*** | .927 | .891 | .053 | .059 | .643 |
|  | Positive Coping | 16 | 18.524ns | .996 | .993 | .020 | .031 | .826 |
|  | Negative Coping | 19 | 45.521*** | .949 | .925 | .058 | .056 | .589 |
|  | Burnout*Engagement | 10 | 7.098^ns^ | 1.000 | 1.074 | .000 | .025 | .498 |
| United Kingdom | Burnout | 78 | 174.078*** | .962 | .949 | .063 | .047 | .905 |
|  | Engagement | 40 | 57.665** | .980 | .973 | .038 | .049 | .770 |
|  | Social Support | 61 | 151.14*** | .938 | .908 | .069 | .054 | .822 |
|  | Positive Coping | 16 | 44.711*** | .971 | .948 | .076 | .050 | .808 |
|  | Negative Coping | 19 | 52.231*** | .963 | .945 | .075 | .050 | .682 |
|  | Burnout*Engagement | 10 | 26.359^ns^ | .936 | .865 | .072 | .047 | .621 |
| United States of America | Burnout | 78 | 156.209*** | .970 | .960 | .056 | .042 | .917 |
|  | Engagement | 40 | 47.074^ns^ | .993 | .990 | .024 | .035 | .773 |
|  | Social Support | 61 | 125.261*** | .959 | .939 | .058 | .049 | .797 |
|  | Positive Coping | 16 | 31.705** | .986 | .975 | .056 | .041 | .819 |
|  | Negative Coping | 19 | 21.018^ns^ | .998 | .997 | .018 | .033 | .683 |
|  | Burnout*Engagement | 10 | 18.9** | .955 | .906 | .053 | .038 | .672 |
| Finland | Burnout | 78 | 229.059*** | .953 | .937 | .074 | .050 | .903 |
|  | Engagement | 40 | 71.341*** | .970 | .959 | .047 | .048 | .741 |
|  | Social Support | 61 | 151.112*** | .939 | .909 | .064 | .052 | .757 |
|  | Positive Coping | 16 | 17.236^ns^ | .999 | .998 | .015 | .024 | .807 |
|  | Negative Coping | 19 | 27.669^ns^ | .992 | .988 | .036 | .036 | .685 |
|  | Burnout*Engagement | 10 | 8.904^ns^ | 1.000 | 1.016 | .000 | .031 | .645 |
| Serbia | Burnout | 78 | 233.898*** | .949 | .932 | .070 | .053 | .901 |
|  | Engagement | 40 | 131.381*** | .930 | .904 | .075 | .062 | .781 |
|  | Social Support | 61 | 251.531*** | .922 | .883 | .087 | .077 | .107 |
|  | Positive Coping | 16 | 76.254*** | .927 | .872 | .096 | .060 | .529 |
|  | Negative Coping | 19 | 31.028** | .985 | .978 | .039 | .022 | .619 |
|  | Burnout*Engagement | 10 | 21.064** | .957 | .909 | .052 | .040 | .640 |
| Taiwan & Macao | Burnout | 78 | 190.567*** | .978 | .971 | .044 | .031 | .956 |
|  | Engagement | 40 | 133.987*** | .962 | .948 | .056 | .039 | .764 |
|  | Social Support | 61 | 241.156*** | .931 | .897 | .062 | .050 | .393 |
|  | Positive Coping | 16 | 64.107*** | .980 | .965 | .063 | .043 | .883 |
|  | Negative Coping | 19 | 40.612*** | .987 | .980 | .039 | .031 | .621 |
|  | Burnout*Engagement | 10 | 15.559^ns^ | .988 | .975 | .027 | .030 | .730 |

(***p<.001, **p<.01, *p<.05, ns – non significant)
